# Phage Annotation Guide: Guidelines for Assembly and High-Quality Annotation

**DOI:** 10.1089/phage.2021.0013

**Published:** 2021-12-16

**Authors:** Dann Turner, Evelien M. Adriaenssens, Igor Tolstoy, Andrew M. Kropinski

**Affiliations:** ^1^ Department of Applied Sciences, Faculty of Health and Applied Sciences, University of the West of England, Bristol, United Kingdom.; ^2^ Quadram Institute Bioscience, Norwich, United Kingdom.; ^3^ Viral Resources, National Center for Biotechnology Information, U.S. National Library of Medicine, Bethesda, Maryland, USA.; ^4^ Department of Food Science, and University of Guelph, Guelph, Ontario, Canada.; ^5^ Department of Pathobiology, University of Guelph, Guelph, Ontario, Canada.

**Keywords:** bacteriophages, phages, genome annotation, annotation guide, genomics, phage taxonomy

## Abstract

All sequencing projects of bacteriophages (phages) should seek to report an accurate and comprehensive annotation of their genomes. This article defines 14 questions for those new to phage genomics that should be addressed before submitting a genome sequence to the International Nucleotide Sequence Database Collaboration or writing a publication.

## Introduction

Comprehensive and accurate genome annotations and critical assessment of genome completeness are crucial facets for the genome sequencing of all organisms. For bacteriophages, the accuracy of annotation has never been more important. The increasing levels of antibiotic resistance in many bacterial nosocomial pathogens have renewed interest in the exploitation of bacterial viruses as therapeutic^1^ and biocontrol agents^2^ and in the study of the molecular mechanisms underpinning productive infection.^3^ Similarly, our understanding that prophages can influence the fitness, phenotype, and global metabolism of the host lysogen necessitates careful identification and annotation of proviral regions within bacterial genomes.^4,5^

The sequencing of phage genomes allows for the delineation of both close and distant relationships within the wider population of phages. However, for any such assessment to be accurate it needs to rely on the diligent annotation of the genome using both automated methods and manual curation. Annotation is not simply about the identification of open reading frames (ORFs) and the putative function of protein-coding genes but should include, in scope, the identification of other functional elements including transfer RNAs (tRNAs), noncoding RNAs, promoters, and terminators.

Above all, the phage biologist should be aware that errors in assembly can lead to mistakes in annotation that can cause the propagation of inaccuracies in the extant sequence databases.

Metagenomics and viromics methods and analyses rely heavily on high-quality genomic databases and annotations to situate metagenome-derived genomes in sequence space. A small error in a sequence database, whether it concerns the length of a protein, an incomplete genome, or an incorrect functional gene annotation can lead to inaccurate interpretations of rapidly increasing sets of metagenomic data.

Three of the authors of this article are members of the Bacterial Viruses Subcommittee of the International Committee on Taxonomy of Viruses (ICTV).^6^ As such, they are committed to assuring that the sequences of phages submitted to the primary International Nucleotide Sequence Database Collaboration (INSDC) databases (GenBank/EMBL/DDBJ)^7^ are of high quality, and that the publications that derive from these submissions are complete and accurate in their annotation and taxonomy. Lamentably, as more people become involved in bacteriophage research, and phage sequencing becomes more high-throughput and automated, we are observing a significant increase in problems.

These include genomes described as circular, chimeric, and incomplete genomes, genomes in which terminal repeats are found in the middle of the sequence, frame shift assembly errors, as well as poorly or incorrectly described gene products.

**FIG. 1.**
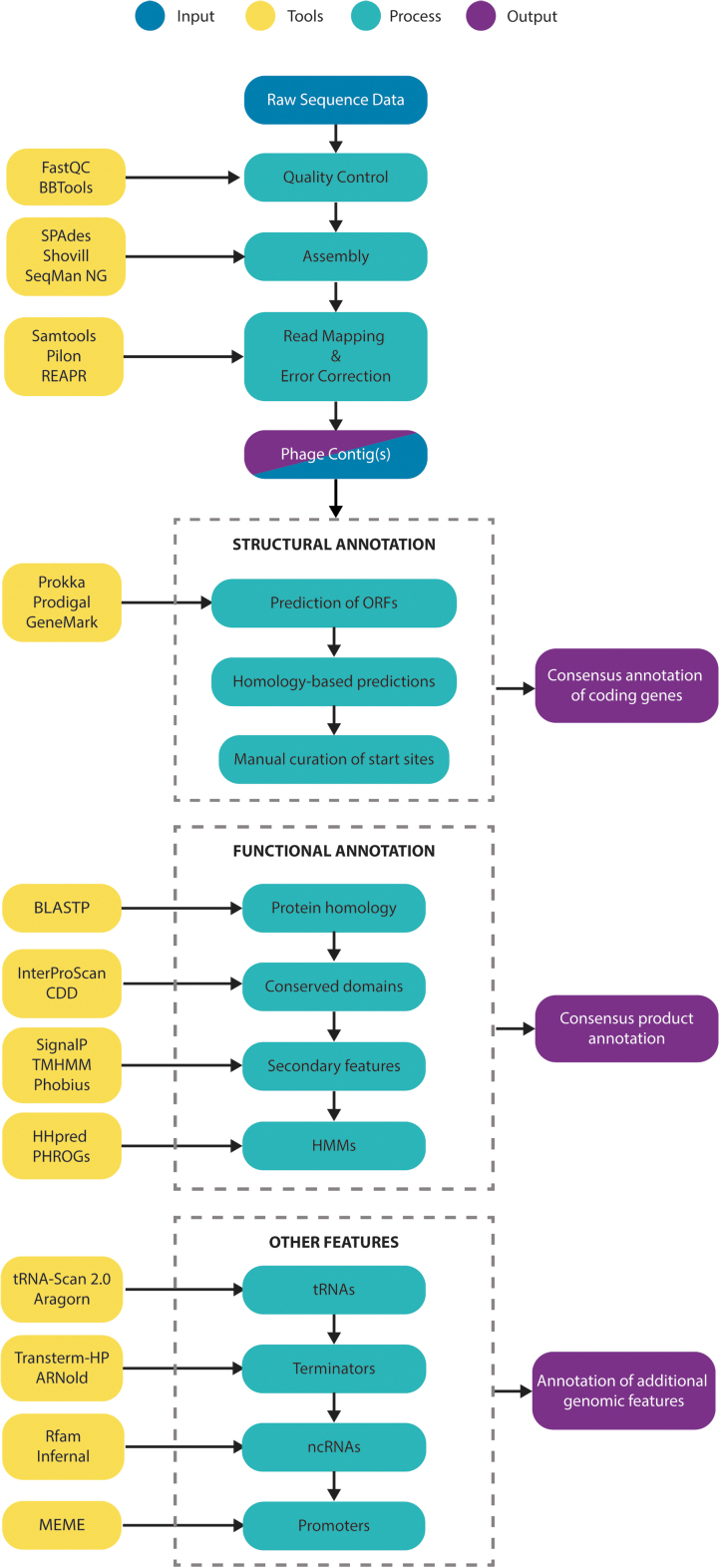
A recommended workflow for the annotation of structural, functional, and other features in assembled bacteriophage genome sequences. Examples of recommended tools for processes are detailed in yellow, but they do not represent an exhaustive list. CDD, Conserved Domains Database; HMMs, Hidden Markov Models; ncRNAs, noncoding RNAs; ORFs, open reading frames; PHROGs, Prokaryotic virus Remote HOmologous Groups; tRNAs, transfer RNAs.

Herein, we describe a set of questions to help phage neophytes ensure that their genome assemblies and annotations are of sufficient quality to be sustainable in the long term. We cover guidelines for assembly, structural and functional annotation. High-quality, well-annotated genomes are an essential tool for both basic and applied research and they provide the basis for the identification and annotation of related genomes. We describe some of the available tools and appropriate approaches, based on both web graphical user interfaces and the command-line. An overview is presented in Figure 1. For a detailed stepwise command-line approach, est genome assembly, and annotation, we refer the reader to the complementary article “Phage Genome Annotation: where to begin and end” by Shen and Millard in this issue.

## Question 1: How Was the Genome Sequenced?

DNA sequencing may occur in one's laboratory, in a centralized “core facility” or by use of commercial providers. When approaching the latter two, it is important to make them aware of the possibility of terminal redundancies and query the use of Nextera (or other “tagmentation”) kits for library preparations, which can result in loss of the genome termini (https://phagesdb.org/blog/posts/26).^8,9^

If one has chosen to employ Illumina, Oxford Nanopore Technology (ONT), or PacBio sequencing, we recommend aiming for between 25 and 100 × coverage. Hundred- or thousand-fold over-coverage will generally not improve your assembly, is unnecessarily expensive, and may result in assembly errors.^9–12^ In comparison to Illumina, there are relatively few reports of phages sequenced solely by ONT or PacBio sequencing. However both ONT and PacBio could be applied for the detection of modified nucleotides or for phages shown to be refractory to conventional sequencing approaches.^13^

Unlike bacterial genomes, where a reference-based assembly can be employed, phage genomes are best assembled *de novo*. A variety of genome assembly software is available for this purpose. In our experience, SPAdes^14^ or Shovill (https://github.com/tseemann/shovill) performs well with *de novo* assembly of phage genomes (with Shovill an Illumina-optimized wrapper for SPAdes that includes subsampling procedures). Command-line instructions for the use of SPAdes are provided by Shen and Millard (supplementary protocol). Alternatively, the commercial programs SeqMan from DNASTAR Ultra (Madison, WI) and CLC Workbench (QIAgen) also work well.

All of these programs can also incorporate longer reads from PacBio and ONT sequencing to generate hybrid assemblies; however, for the majority of bacteriophages, long-read technologies and hybrid assemblies will not be required to assemble the complete genome. For a detailed assessment of the application of assemblers on Illumina data at different depths of sequencing, we refer the reader to Rihtman et al.^9^ All assemblers that utilize short-read (read Illumina) data inevitably generate low coverage and spurious homopolymeric contigs that should be filtered out.

Assembly metrics should be assessed. How many contigs have been assembled? What is the mean, minimum, and maximum length of the assembled contigs? What is the depth of coverage across the (phage) contig(s) and is it consistent? Differences in coverage between contigs often point toward bacterial contamination, the presence of prophages induced at low levels, and/or the existence of multiple phages in the sample. One should keep in mind that the purification process will influence the presence of host DNA—without a very extended DNase and RNase step, PEG purified preparations generally will include some contaminating host DNA and potentially induced prophages. These assembly statistics can be determined by using programs such as Qualimap^15^ and QUAST.^16^

The contig(s) should also be inspected for duplicated regions, which is easily checked for using dotplots (e.g., Gepard^17^) or BLASTN, as contigs may be extended to more than 100% of the phage genome length.

Once the contig has been assessed, a critical step is to map the individual reads back to the genome/contig by using appropriate read alignment software, for example, BWA-MEM,^18^ Minimap2,^19^ Bowtie2,^20^ and samtools.^21^ Once done, the mapping should be inspected to identify (1) regions where the paired reads significantly violate the expected distance between paired reads or in the mapped orientation of reads and (2) to identify whether there are any regions of excess or low coverage. Assemblies may require error correction with polishing tools, a process described by Shen and Millard in step 8 of the supplementary protocol.

## Question 2: Is the Genome Complete?

The ICTV-acceptable interpretation of the complete genome coverage of bacteriophages is that you have the complete unique sequence of your virus. Phage genomes come in a variety of configurations that have implications for assembly and downstream processing. The most common configurations for isolated tailed phages are circularly permuted (CP), terminally redundant (TR) (defined ends with terminal repeats), or cohesive ends (defined ends with short single-stranded overhangs). Other types are possible, such as ssDNA circular genomes or even dsRNA segmented genomes, which will not be discussed here.

To be considered as complete, in the case of phages that possess TR ends, this means that the sequence includes at minimum one of the redundancies.

PhageTerm^22^ accessible as part of the Galaxy Tool Shed^23,24^ or via the command-line (see Shen and Millard, step 11 of the supplementary protocol) can be used to provide a prediction of the type of genome termini present, but these predictions should be verified by using run-off Sanger sequencing. For more information of phage termini, we recommend that you consult (Chung et al.^25^ and https://phagesdb.org/documents/).

## Question 3: Does the Sequence Contain Ambiguous Bases or Potential Frameshifts?

Ambiguous bases, often denoted by the International Union of Pure and Applied Chemistry (IUPAC) code of N (any base), should be avoided. These discrepancies should be resolved by targeted Sanger sequencing and can be identified by any online resources that count bases.

Three varieties of frameshifts exist—sequencing/assembly errors, programmed frameshifts, and introns. Should a homologous phage exist then at the DNA level BLASTX can often be used to identify frameshifts. Programmed frameshift is often discovered as a result of in-depth proteomic analyses. In the case of coliphage lambda, tail assembly presents an example of a programmed translational frameshift, resulting in the gpGT protein,^26^ a feature also uncovered in tails of other bacteriophages.^27,28^ Another common example is the presence of two major capsid proteins (10A/10B) in *Escherichia* phage T7.^29^

## Question 4: Is the Genome Assembly Co-linear with That of Closely Related Genomes?

**FIG. 2.**
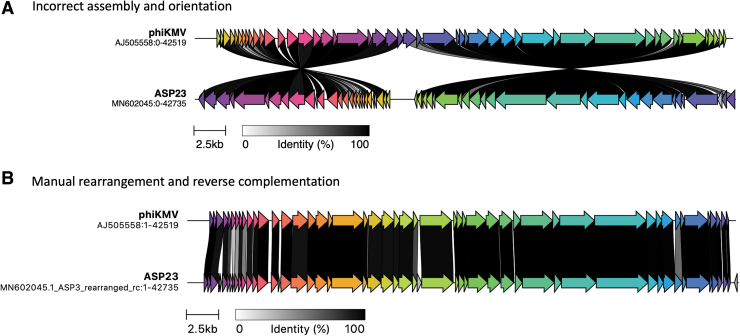
Genome CDS comparison between Pseudomonas phages phiKMV (AJ505558) and vB_PaeP_ASP23, short ASP23 (MN602045) using clinker. Homologous CDSs are in the same color and linked through gray bars with the percentage amino acid identity, as indicated in the legend. **(A)** Direct comparison and visualization of GenBank records (accessed October 25, 2021). **(B)** Manual rearrangement of the ASP23 genome in TextEdit on Mac and reverse complementation with the Sequence Manipulation Suite,^114^ followed by reannotation with Prokka.^89^ CDS, coding sequence.

Circularization of phage genomes is a common artefact of assembly. No dsDNA tailed phage genome is truly circular when packaged in the capsid, but it can be CP or have terminal repeats that result in an *apparently* circular assembly. With CP genomes it is often easy to convince oneself that the genome is circular^30^ but this is not the case. In addition, during the assembly of TR genomes, the redundancies may end up being located internally within the contig (Fig. 2).

If your phage genome is CP, then the choice of “beginning” and “end” is an arbitrary one. If your phage is related to one already present in the National Center for Biotechnology Information (NCBI), then your chromosome should be made co-linear with that of the reference genome or the “type virus.” This can be readily ascertained from the BLASTN output or using a visualization tool such as progressiveMauve^31^ or clinker (https://github.com/gamcil/clinker).

Without a homologous genome in the database, and without defined ends, we suggest that you choose the “beginning” in an intergenic region that separates operons. Inspiration can be found by using more distant relatives, for example after the *rIIB* gene for T4-like phages.^32^ For siphoviruses, past convention has been to open the assembly at the start codon of the small terminase subunit.

By ensuring that the genome begins at experimentally defined termini, or opening the genome at a specific gene, one avoids the common problem of an ORF being split across the ends of the contig and enables easier interpretation of visual pairwise comparison of genomes using visualization tools.

Methods for the identification of genome termini using PhageTerm, and the reordering of phage genomes are detailed in Shen and Millard (supplementary protocol, steps 11 and 12).

## Question 5: How Was the Genome Annotated?

Annotation can be sub-divided into structural annotation, the identification of coding sequences (CDSs), and functional annotation, the identification of gene products. Here, we will distinguish between annotation with visual oversight (manual annotation) and without oversight (auto-annotation). Although the SEA-PHAGES (Science Education Alliance-Phage Hunters Advancing Genomics and Evolutionary Science; https://seaphages.org/) program employs the Windows-based program DNA Master^33^ to annotate bacteriophage genomes, many use auto-annotation as the first step toward the complete analysis of our phage genomes.

Unfortunately, most of the programs that we use were designed for the annotation of bacterial genomes that tend to possess larger ORFs than those of their viruses. This means that many, particularly smaller, CDSs may have been missed. To rectify this problem, one needs freeware such as Artemis^34^ (https://www.sanger.ac.uk/tool/artemis/), DNA Master, UGENE,^35^ or commercial programs such as DNASTAR or Geneious (Table 1) to examine the DNA sequence for ORFs and potential CDSs, considering all ORFs >75 nt.^36^

**Table 1.** Software for Phage Genome Annotation

Program	Usage	Source	Reference
Prokka	MAC, Linux/Galaxy	https://github.com/tseemann/prokka	^89^
RAST	WEB	https://rast.nmpdr.org/	^90,91^
PATRIC	WEB (RAST)	https://www.patricbrc.org/	^92^
DFAST	WEB	https://dfast.ddbj.nig.ac.jp/	^93,94^
Phage-specific Galaxy instance	WEB	https://cpt.tamu.edu/	^95^
UGENE	WIN, MAC, Linux	http://ugene.net/	^35^
Phage Commander	MAC, Linux	https://github.com/mlazeroff/PhageCommander	^49^
multiPhATE	MAC, Linux/Galaxy	https://github.com/carolzhou/multiPhATE2	^96^
DNA Master	WIN	https://phagesdb.org/DNAMaster/	^33^
DNASTAR (COM)	WIN, MAC	https://www.dnastar.com/	^97^
Geneious (COM)	WIN, MAC	https://www.geneious.com/	^98^
VIGA	MAC, Linux	https://github.com/EGTortuero/viga	

COM, commercial software; MAC, Mac computer; WEB, Internet resource; WIN, Windows.

Phage genomes possess a high coding capacity; therefore, one might expect to see small gaps between CDSs or small overlaps. Large gaps should immediately alert you to potentially missing genes and should be manually verified, though some phages such as *Escherichia* phage rV5^37^ do possess wastelands with no apparent genes.

**FIG. 3.**
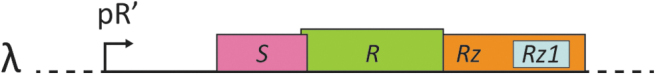
An example of an embedded gene—Rz1 within R of phage lambda, adapted from Rajaure et al.^115^

There are three unusual cases that the researcher should keep in mind while annotating their genomes. These include CDSs within CDSs, frameshifts (e.g., GpG, GpGT tail assembly chaperones, as mentioned earlier), and the presence of introns and inteins. Although embedded genes are rare among members of the class *Caudoviricetes*, there are some notable instances where they can be found.^38–40^ One of the best examples is in the lysis cassette of Escherichia virus λ where genes *S*, *R*, *Rz*, and *Rz1* encode the holin, endolysin, i-spannin, and o-spannin, respectively (Fig. 3). We recommend that you employ extreme caution in recording embedded genes in your genome annotation.

**FIG. 4.**
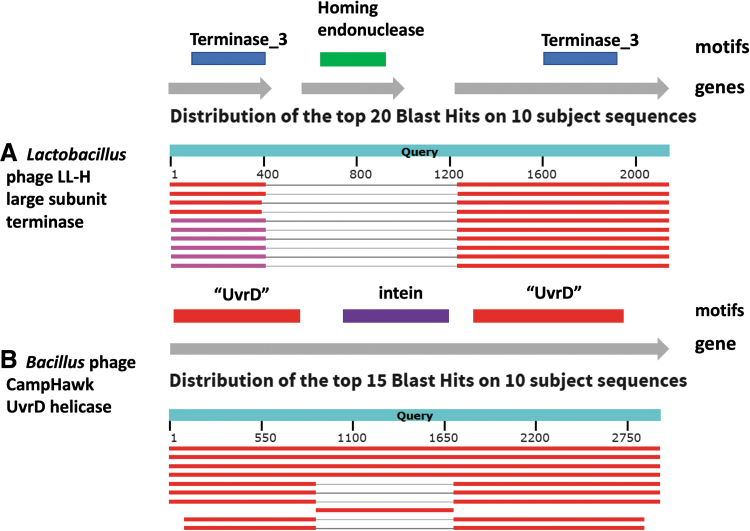
**(A)** BLASTX analysis of the Lactobacillus phage LL-H large subunit terminase gene (NC_009554.1, 1647..3785) in which it appears that this region contains three ORFs, two of which on translation show homology to TerL proteins and one similarity to a homing endonuclease. **(B)** BLASTX analysis of Bacillus phage CampHawk (NC_022761.1, 118288..121224), which encodes a UvrD-like helicase with an intein.^116^ A nearly identical sequence is found in Bacillus phage vB_BsuM-Goe2, but not in Bacillus phage SP8. The CampHawk helicase contains 978 amino acids, whereas its homolog in SP8 possesses only 703 residues.

The presence of inteins^41^ or introns is usually suspected if the size of the gene is significantly different from that of the expected protein, or if the gene is split across multiple ORFs (Fig. 4). Introns are relatively common in phages with large genomes, particularly those that infect *Campylobacter* and *Staphylococcus*, but size should not be used as an excuse not to examine for their presence since they have been found in many differently sized viruses.^42–45^ The intron possesses ribozyme activity, which splices itself out of the messenger RNA (mRNA), resulting in the mature mRNA, which is translated into a protein. This region may encode a homing endonuclease, which is the case with *Lactobacillus* phage LL-H (Fig. 4A).

**FIG. 5.**
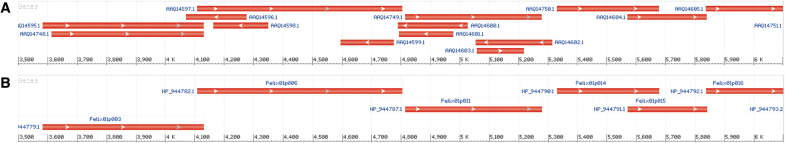
An example of an over-annotated genome. **(A)** Bacteriophage Felix 01 GenBank record (AF320576), graphical view of the region 3500..6100. **(B)** RefSeq curated record for Enterobacteria phage Felix 01 (NC_005282.1) graphical view of the region 3500..6100. Annotated coding sequences are depicted as red rectangles, with arrows denoting presence on the forward or reverse strand.

Including all possible internal and overlapping ORFs as CDSs can lead to over-annotation of a genome (Fig. 5). In the case of phage Felix01, the originally annotated sequence of all the genes corresponded to 113.7 kb, that is, a coding capacity of 1.32. When the annotation was curated as part of the NCBI Reference Sequence validation process,^46^ the coding capacity was reduced to 0.90 (77.6 kb) after the removal of spurious ORFs—a value that is near the norm for phages belonging to the class *Caudoviricetes*.

## Question 6: Were CDS Start Sites Curated?

Most tailed phages use the general bacterial translation table (table 11), with ATG and GTG being the most common start codon, but also a set of alternative initiation codons (TTG, CTG, ATT, and ATC).^47,48^ These alternative start codons are not always recognized by automated gene calling software, and differences in gene calling between programs can now be assessed with the new tool PhageCommander.^49^ Best practices, therefore, include the manual curation of the start codons to optimize coding density and ensure that ribosome binding sites (RBS, or Shine–Dalgarno sequences) precede each start codon.

**FIG. 6.**
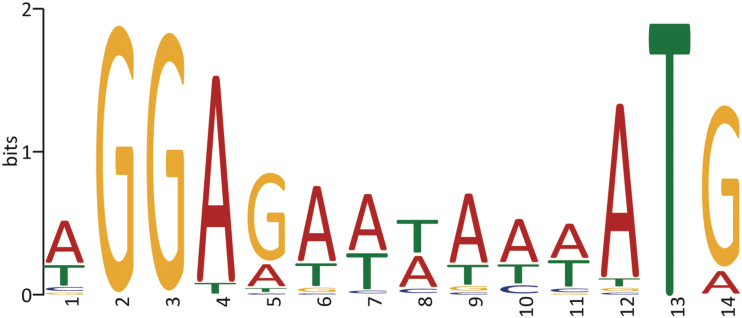
Sequence logo of a statistically over-represented motif (Shine–Dalgarno sequence or ribosome-binding site) identified by using MEME from 103 bp sequences encompassing the predicted start codon and upstream region.

A meta-analysis of all known viruses showed the presence of an RBS in the form of AGGAGG (or 4mer/5mer variations of this sequence) in more than 50% of the CDSs of all bacteriophages.^50^ One way of assessing the presence or absence of ribosome binding sites is to extract sequences that encompass 100 bp upstream of the ORF and the predicted start codon for analysis using MEME^51^ (Fig. 6).

## Question 7: How Were the Functions of the Gene Products Identified?

There are now a wealth of tools to enrich the functional annotation of gene products beyond that obtained by BLASTP alone, which in many cases appears to be the only tool used. Moreover, an over-reliance on automated BLASTP functional assignments can result in the miss-annotation of proteins, a problem that has the potential to propagate errors through the INSDC databases. An appropriate approach is to look for some manner of consensus across a number of different annotation methods. In addition to BLASTP and Position Specific Iterated BLAST (PSI-BLAST), we recommend the use of InterPro,^52^ Batch Web CD-Search Tool,^53^ and HHpred (or the command-line instance HHsuite).^54^ Another useful sequence feature to scan for are transmembrane domains, which can be readily identified by using Phobius^55^ and TMHMM.^56^

Please beware of calling a protein a DNA polymerase based on no or poor quality evidence since this leads to miss-annotation creep and database poisoning, which is far worse than designating a DNA polymerase as a “hypothetical protein.” Again please exercise caution by not over-relying on BLASTP hits.

In addition to the recommendations outlined here, Shen and Millard provide routes to customize the automated annotation of gene products with Prokka that implement the Prokaryotic virus Remote HOmologous Groups (PHROGS) and custom-tailed phage databases for improved inference of function (supplementary protocol, steps 14 and 15).

### A note on standardized terminology

One of the most common proteins encoded by T4-like phages is RIIA, yet an examination of homologs in NCBI reveals names that vary from “rIIA protector from prophage-induced early lysis,” “membrane-associated affects host membrane ATPase,” and “rIIA protein,” to “hypothetical protein,” “unnamed protein product,” and “protein of unknown function.” The last three annotations are examples of poor annotation since the functions of homologs of this protein are clearly known.

Please note that “phage hypothetical protein” is redundant since all of your viruses' proteins are “phage proteins.” If no function can be predicted, the term “hypothetical protein” should be used as the product qualifier value.

Product names such as “UboA,” “Mcp,” “hypothetical protein SA5_0153/152,” “ORF184,” “gp200,” “RNAP1,” “32 kDa protein,” and “hypothetical protein HY02_082” should not occur in your annotated genome because they do not mean anything to the casual, or indeed even the informed, reader. The use of “gp” (gene product) is common but should be discouraged since gp200 describes radically different proteins in *Listeria*, *Enterococcus*, *Mycobacterium*, *Rhodococcus*, *Sphingomonas*, *Pseudomonas*, *Bacillus*, and *Synechococcus* phage genomes. If you want to relate it to an existing protein, you can add the information as a note to the annotation, for example, /note = “similar to gp43 of Escherichia phage T4.”

Unless you are a bioinformatician or biostatistician, or an expert in a specific protein family, be very conservative in recording a protein function from “BLAST hits.” Could you convince your grandmother? If not, list it as a “hypothetical protein.” If you have some homology or motif data that suggest that it may be a DNA polymerase, describe it as a “hypothetical protein” and then add an “evidence qualifier” (see, https://www.ncbi.nlm.nih.gov/genbank/evidence/) such as /inference = “protein motif DNA_pol_B_2 (PF03175).” Do not name the gene product “putative DNA polymerase.”

We would recommend that you consult the UniProt Knowledgebase (UniProtKB^57^), which is a manually curated and reviewed information database on proteins (https://www.uniprot.org/) and ViralZone^58^ (https://viralzone.expasy.org/) when assigning names to gene products.

## Question 8: How Did You Screen for Integrases/Recombinases, Toxins, and Antibiotic-Resistance Genes?

Since many phages are isolated as potential therapeutic agents, the presence of indicators of a temperate lifestyle and the carriage of toxin genes would preclude their use.

Predictions of temperate lifestyles from genomic data were traditionally done by manual scanning of the predicted proteins for lysogeny-related genes (integrases/recombinases/transposases). Automated lifestyle predictions can be done with PhageAI,^59^ PHACTS,^60^ or BACPHLIP,^61^ which are based on the presence of conserved domains, or in the case of phage.ai through machine learning and natural language processing. As with all automated predictions, use caution when the evidence scores are low.

Although there is plenty of evidence that bacterial antibiotic-resistance genes (ARGs) genes can be disseminated by phage-mediated transduction,^62^ and indeed viromes have been demonstrated to have associated ARGs,^63–66^ the identification of these elements in phage genomes should be treated with extreme caution and a rare occurrence.^67^ The Comprehensive Antibiotic Resistance Database^68^ (https://card.mcmaster.ca) is a vital resource, but the results should be treated with great skepticism unless predicted functions have been experimentally verified. Another resource for the identification of ARGs is AMRFinderPlus, developed at the NCBI.^69^

The presence of toxin-encoding genes in the phage genome immediately precludes the use of that phage for therapeutic purposes. A number of Internet resources will assist you in determining whether your phage carries a toxin gene (Table 2). Once again use caution when interpreting the results.

**Table 2.** Internet Resources for Toxin Screening

Program name	URL	Comment	Reference
VirulenceFinder 2.0	https://cge.cbs.dtu.dk/services/VirulenceFinder/	Not all pathogens	^99^
VirulentPred	http://203.92.44.117/virulent/submit.html		^100^
DBETH	www.hpppi.iicb.res.in/btox/cgi-bin2/svm.cgi?name=svm	Single sequence	^101^
T3DB	www.t3db.ca/biodb/search/target_bonds/sequence	Single sequence	^102^
VFDB	www.mgc.ac.cn/VFs/search_VFs.htm	Single sequence	^103^

Further considerations for requirements for the annotation of therapeutic phages are discussed by Shen and Millard.

## Question 9: Were Putative Promoters, Terminators, and Other Elements Identified?

Though not strictly necessary in genome submissions or publications, some authors choose to screen their genomes for host- or phage-RNA polymerase-dependent promoters, and ρ-independent terminators. To our knowledge, no one has yet analyzed ρ-dependent terminators.^70^ Since, in the absence of RNA-seq data, these elements are all theoretical, we recommend that authors err on the side of caution when presenting data. Toward this end, promoters and terminators should only be added to the annotation if they are at the 5′ or 3′-end of genes, respectively, or in the intergenic regions.

Promoters employing RpoD (Sigma70)-dependent host RNA polymerase can be identified by using a variety of online sites (Table 3).

**Table 3.** Internet Resources for Screening Your Phage Genome for Promoters

Program name	URL	Comment	Reference
Bacterial promoters			
PromoterHunter	www.phisite.org/main/index.php?nav=tools&nav_sel=hunter	Part of phiSITE	^104^
PhagePromoter	https://galaxy.bio.di.uminho.pt/?tool_id=get_proms&version=0.1.0		^105^
Genome2D (Prokaryote Promoter Prediction)	http://genome2d.molgenrug.nl/g2d_pepper_promoters.php	Part of PePPER	^106^
SAPPHIRE	https://sapphire.biw.kuleuven.be/		^107^
Phage promoters			
PHIRE	https://www.biw.kuleuven.be/logt/PHIRE.htm	Very slow; WIN	^108^
MEME	https://meme-suite.org/meme/	Part of the MEME Suite	^51^
STREME	https://meme-suite.org/meme/tools/streme	Part of the MEME Suite	^109^

The host housekeeping (RpoD-dependent) promoters in Gammaproteobacteria possess the consensus sequence TTGACA[N15-19]TATAAT.^71^ We recommend that you consult the literature for the latest consensus sequence for the bacterium of interest.^72^ One of the authors (A.M.K.) only records sequences that differ by two nucleotides or less from the consensus. It is appropriate, but not obligatory, to include the transcriptional start site (+1). If your putative promoter includes an AT-rich upstream promoter DNA element,^73^ please include it. If not, trim to the consensus.

Factor ρ-independent terminators can be identified by using a variety of online tools (Table 4) and should be trimmed to remove sequences 5′ of the uploop and 3′ of the final thymidylate residue. In this case, we recommend that you only record terminators that display a Gibbs free energy (ΔG) equal to or lower than −10 kcal/mol.^74^

**Table 4.** Internet Resources for Screening Your Phage Genome for ρ-Independent Terminators

Program name	URL	Comment	Reference
Genome2D (Transcription Terminator Prediction)	http://genome2d.molgenrug.nl/g2d_pepper_transterm.php	Part of PePPER	^106^
ARNold	http://rssf.i2bc.paris-saclay.fr/toolbox/arnold/	Nice output	^110^
FindTerm	www.softberry.com/berry.phtml?topic=findterm&group=programs&subgroup=gfindb	Part of SoftBerry suite	^111^
iTerm-PseKNC	http://lin-group.cn/server/iTerm-PseKNC/		^112,113^

Lastly, tRNAs are a common structural element, particularly in large phage genomes.^75^ The two programs that we recommend are tRNAscan-SE at http://lowelab.ucsc.edu/tRNAscan-SE/^76^ and ARAGORN at www.ansikte.se/ARAGORN/.^77^ Please note that bacterial and bacteriophage tRNAs possess a CAA triplet at their 3′-termini, which are sometimes missing from auto-annotated genomes.

## Question 10: Does the Sequence Represent a Phage Isolate, Prophage, or a Metagenome-Derived “Envirophage”?

Unless specified within the annotation submitted to the INSDC, it is often impossible for the reader to discern whether a deposited sequence represents a lytic or temperate phage isolated by using a specific host, an induced prophage only known from its bacterial genome coordinates, or a sequence derived from metagenomic analyses (envirophage). Before submission, we recommend that phage biologists look in detail at the available source feature keys (https://www.insdc.org/documents/feature-table#7.3).

The qualifier “/proviral” can be used within the source feature key to denote that the sequence has been obtained from an induced prophage using the bacterial genome sequencing data. In addition, the qualifier “/host” can be used to inform the reader which host strain a prophage was induced from. The qualifier “/lab_host” can be used to denote the host stain used for isolation and/or propagation. Additional information, for example “/isolation_source” or “/country,” could also be made included to expand the available information.

Similarly, for “envirophages,” the qualifier/environmental_sample indicates that the genome sequence was derived, not from an isolate, but from a bulk environmental nucleic acid sample, without culturing. This qualifier should always be used in conjunction with the /isolation_source qualifier to indicate the type of sample the sequence was derived from. Phage genomes with these qualifiers will get an “ENV” tag in databases.

## Question 11: Have You Chosen a Realistic and Useful Phage Name and Locus Tag?

Here, we must reintroduce the importance of choosing a “good” name for your phage. We strongly recommend that authors consult Adriaenssens and Brister^78^ together with NCBI (https://www.ncbi.nlm.nih.gov/labs/virus/vssi/#/), Phage Name Check (www.phage.org/phage_name_check.html), and CPT Phage Name Search (https://cpt.tamu.edu/phage-registry/) to assure that the phage name is unique. Although simple alphanumerically names such as P22 and T4 used to be the norm, today most people are following the lead of the SEA-PHAGES program^79,80^ and opt for a common name such as Abrogate, Adjutor, and Adonis.

These names greatly assist in the creation of new genera, and binomial species names,^81^ which often use the unique part of exemplar phage names for inspiration. In addition, “Locus_tags are identifiers that are systematically applied to every gene in a genome. These tags have become surrogate gene names by the biological community.” (https://www.ncbi.nlm.nih.gov/genomes/locustag/Proposal.pdf) We recommend that you employ the unique part of the phage name as part of the locus tag. In addition, if your group plans on submitting many different sequences it may be worth considering adding a common set of letters to the locus tag for recognition purposes.

## Question 12: Does the Submitted Sequence Contain Irrelevant Material?

Before submission to one of the three major INSDC databases, you want to clean up your submission to remove irrelevant material. Please check that the genome is indicated as linear, not circular. The “Definition” line should read “host genus + phage + name,” for example, Proteus phage Mydo. Do not include host species name, isolate, chromosome, DNA, or genome. Many of the automated annotation programs leave evidence of their use, such as RAST “db_xref = "SEED:fig|6666666.271554.peg.1”

All local/temporary identifiers that cannot be found by external users on the web should be deleted. This can be easily accomplished in using a text editor such as Notepad (Windows), TextEdit (Mac), or the more powerful freeware tool Notepad++ (Windows).

## Question 13: Is the Taxonomy That I Have Assigned to This Phage Correct?

Although a detailed discussion of phage taxonomy is outside of the scope of this article, some general thresholds can be reiterated.^82^ If your phage exhibits ≥95% sequence similarity (by VIRIDIC^83^ or for BLASTN,^84^ coverage multiplied by identity), then it represents a new strain of an existing phage species. If it exhibits <95% but ≥70% sequence similarity it represents the first isolate of a new species in an undefined or existing genus. The delineation of subfamily or family-level relationships requires more careful inspection, including pangenome analysis and the inference of single gene or concatenated/partitioned signature gene phylogenies. In the case of confusion or questions, please consult the appropriate member of the Bacterial Viruses Subcommittee of ICTV (https://talk.ictvonline.org/information/members-606089945/w/members/441/bacterial-viruses-subcommittee).

## Question 14: Should I Update My Database Submission When New Data Renders It Dated?

Data do not rest, nor does the phage research community. Alongside homology searches of CDSs, it is worthwhile reading the published literature associated with closely related phages. Often, this will reveal experimental evidence regarding the identification of phage structural proteins, such as from mass spectrometry data. Similarly, any studies of phage proteins encompassing cryo-electron microscopy, crystallography, or nuclear magnetic resonance can enrich your annotation given an appropriate level of sequence homology and predicted protein secondary structure. Transcriptomics studies of phage infection may lead to updated information about start codons and location of regulatory elements.

It is important to remember that you “own” your genome sequence and annotation; the INSDC prefers that any updates arise from the original submitter and as such the authors should take responsibility for updating their own records. Please note, that any phage sequence submitted without annotation is automatically marked as “unverified” according to GenBank policy.

## Final Statement

Sequencing, assembling, and annotating a newly isolated phage is a rewarding process and contributes data to our knowledge of the global phage population. However, the value and utility of these data is dependent on a careful, measured, and diligent approach to these processes. It is our hope that the answers to these questions will provide direction and prove useful to the wider community of phage biologists. Similarly, the tools and programs detailed here do not represent an exhaustive list of those available, and new tools are developed all the time. We highly recommend consulting Shen and Millard, who provide additional considerations on these 14 questions as well as a step-by-step guide to phage genome assembly and annotation that employs a number of tools mentioned here.

Happy annotating!

## Further Practical Information Can Be Found In…

Bacteriophages: Methods and Protocols Volume 3^85^:

◦ Chapter 9: Sequencing, Assembling, and Finishing Complete Bacteriophage Genomes^86^
◦ Chapter 11: Analyzing Genome Termini of Bacteriophage Through High-Throughput Sequencing^87^
◦ Chapter 16: Annotation of Bacteriophage Genome Sequences Using DNA Master: An Overview^33^
◦ Chapter 18: Visualization of Phage Genomic Data: Comparative Genomics and Publication-Quality Diagrams^88^

We encourage users to read publications associated with the tools and programs mentioned here and to use the variety of discussion-board platforms available (e.g., SeqAnswers, Biostar, ResearchGate) to search for advice and for trouble-shooting.
